# A Case Report on Aggressive Natural Killer Cell Leukemia in a Pediatric Patient

**DOI:** 10.7759/cureus.25634

**Published:** 2022-06-03

**Authors:** Umberto M Donato, Celeste Shoeleh, Andrew Galligan

**Affiliations:** 1 Pediatric Hematology Oncology, University of South Florida Morsani College of Medicine, Tampa, USA; 2 Pediatric Oncology, Moffitt Cancer Center, Tampa, USA

**Keywords:** ebv, neoplasm, rare presentation, pediatric cancer, ankl

## Abstract

Aggressive natural killer cell leukemia (ANKL) is a rare neoplastic malignancy, especially in pediatric populations with very few cases reported in the literature. It commonly presents with a rapidly declining clinical course and has a median survival of two months. We report the case of a 15-year-old female who presented with fever, hepatosplenomegaly, hemophagocytosis, and disseminated intravascular coagulation (DIC). The patient was ultimately diagnosed with ANKL but died after developing multiorgan failure and DIC prior to the initiation of any treatment. In this case report, we review and discuss the literature concerning the diagnosis and treatment of ANKL in pediatric patients.

## Introduction

Aggressive natural killer cell leukemia (ANKL) is a rare form of natural killer (NK) cell neoplasm characterized by a sudden onset and aggressive clinical course [1]. ANKL is closely associated with Epstein-Barr virus (EBV) and East Asian populations. This rare neoplasm typically presents in patients between 20 and 50 years of age, with a median age of onset of 40 years [2,3]. The most common clinical symptoms include fever, hepatosplenomegaly, hemophagocytosis, and disseminated intravascular coagulation (DIC). However, limited data on clinical presentation and a lack of diagnostic methods for ANKL have resulted in the misdiagnosis and delayed diagnosis of ANKL. Furthermore, even though around 200 to 350 cases of ANKL have been reported in the English literature, only a small fraction of cases have been reported in the pediatric population [3,4].

In addition to being challenging to diagnose, ANKL has a poor prognosis, with a median survival of two to three months post-diagnosis, and no standard treatment options [5]. L-asparaginase-containing regimens, including dexamethasone, methotrexate, ifosfamide, l-asparaginase, and etoposide (SMILE), are often employed but rarely extend survival for more than a year [5,6]. Allogeneic hematopoietic cell transplantation (HCT) has been performed in select patients and has shown to prolong survival and possibly cure some patients, but with very limited success [6].

Here, we describe the case of a young 15-year-old female with ANKL who presented with fever, hepatosplenomegaly, hemophagocytosis, and DIC, as well as fatigue, abdominal pain, and unintended weight loss. We intended to start treatment with a modified SMILE or pegaspargase, gemcitabine, and oxaliplatin (P-GEMOX) regimen but did not begin treatment due to concern of deterioration before a possible HCT. The patient remained unstable and died two weeks post-diagnosis due to multiorgan failure.

## Case presentation

A 15-year-old female with no significant medical history presented with a three-week history of fever, fatigue, nausea, headaches, night sweats, diarrhea, increased urinary frequency, and unintended weight loss. Upon admission, vitals were remarkable for an elevated temperature and pulse along with a decreased diastolic blood pressure (Table 1). During this time, she experienced hepatosplenomegaly, hemophagocytosis, and DIC. Low platelet levels, decreased fibrinogen concentrations, and prolonged prothrombin times were concerning for DIC (Table 2). Abdominal ultrasound showed hepatosplenomegaly with iron deposition and a left lobe liver mass. The laboratory values were found to be hemoglobin of 11.1 g/dL (normal range: 12-16 g/dL), white blood cell (WBC) count of 4.8 × 10^3^/µL (normal range: 4.5-10.5 × 10^3^/µL), platelet count of 118 × 10^3^/µL (normal range: 150-400 × 10^3^/µL), C-reactive protein of 4.96 mg/dL (normal range: 0.01-0.5 mg/dL), and erythrocyte sedimentation rate of 35 mm/hour (normal range: 0-20 mm/hour). In a peripheral smear at presentation, 3% metamyelocytes, 4% monocytes, and 8% lymphocytes were observed. A bone marrow biopsy was performed and was concerning for an EBV infection. Flow cytometry results reported a subset of aberrant CD2+/CD8+/CD56+ NK cells that were seen in the marrow. Biopsy results also showed the presence of intact hematopoietic cells (erythrocytes, leukocytes, or both) within the cytoplasm of phagocytic histiocytes, observations consistent with a diagnosis of hemophagocytosis [7].

**Table 1:** Vital signs of the patient.

	Values at presentation (1/24/21)	Values at progression (2/11/21)	Reference values
Temperature (°C)	37.2	36.3	35.5–37.5
Pulse/minute	103	120	50–100
Respirations/minute	16	20	12–20
Blood pressure (mmHg)	113/66	85/47	114/74
Pulse oxygen (%)	99	94	95–100

**Table 2:** Diagnostic lab values. ALT: alanine transaminase; AST: aspartate transaminase; BUN: blood urea nitrogen; EBV: Epstein-Barr virus; PCR: polymerase chain reaction; DIC: disseminated intravascular coagulation

Diagnoses	Components	Values at presentation (1/24/21)	Values at progression (2/11/21)	Reference values
Thrombocytopenia	Platelets (µL)	81 × 10^3^	58 × 10^3^	142.0–424.0
Anemia	Hemoglobin (g/dL)	7.3	9.2	12.2–16.2
	Hematocrit (%)	24.0	19.5	37.7–47.9
Transaminitis/Liver injury	ALT (U/L)	68	86	5–55
	AST (U/L)	72	328	5–34
Acute renal injury/Failure	Serum creatinine (mg/dL)	2.74	2.74	0.57–1.11
	BUN (mg/dL)	21	39	6–20
	Serum uric acid (mg/dL)	16.2	2.3	1–6
EBV +	EBV PCR (copies)	>1.79 × 10^3^	>1.56 × 10^3^	0
DIC	PTT (seconds)	13.3	34.1	10.3–14.5
	Fibrinogen (mg/dL)	415	156	190–500

Laboratory data revealed thrombocytopenia, anemia, transaminitis, and, consequently, liver damage (Table 2). During the follow-up period, her anemia and thrombocytopenia deepened and her leukocyte count increased approximately threefold (Table 2). Elevated serum creatinine, blood urea nitrogen, and serum uric acid (Table 2) were suspicious for acute kidney injury (AKI). Right cervical lymph node biopsy was then performed and revealed EBV-positive T/NK cell lymphoproliferative disorder of childhood, either lymphoma or chronic active infection. EBV serology was found to be consistent with an acute infection. The presence of viral capsid antigen (VCA) immunoglobulin (Ig)M and VCA IgG without EBNA-1 IgG were suggestive of acute infection [8]. The lymph node biopsy was sent to the National Institutes of Health (NIH) for a second review and revealed a diagnosis of ANKL. Her initial AKI and elevated uric acid were likely multifactorial, including pre-renal and tumor lysis syndrome.

The initial plan was to start treatment with the cyclophosphamide, doxorubicin hydrochloride (hydroxydaunorubicin), vincristine sulfate (Oncovin), and prednisone (CHOP) protocol, which is the standard approach for the majority of hematogenous malignancies. Once the ANKL diagnosis was confirmed, the option of a more aggressive chemotherapy approach such as SMILE or P-GEMOX was discussed. Neither of the regimens was introduced due to concern of decompensation. The patient was consequently started on intravenous dexamethasone (4 mg) twice a day along with low-dose continuous Dilaudid via patient-controlled analgesia. There was a resultant improvement in pain and vital signs. Additionally, blood pressures normalized along with heart rate and respiratory rate significantly improved close to or within reference values for her age. Be that as it may, the disease progressed. WBC, mean corpuscular volume, red cell distribution width, and nucleated red blood cell (RBC) values rose considerably (Table 3). RBC, hemoglobin, hematocrit, and platelet counts continued to decrease noticeably (Table 3). The patient once again remained critically unstable and passed away due to multiorgan failure two weeks post-ANKL diagnosis.

**Table 3:** Complete blood count findings.

	Values at presentation (1/24/21)	Values at progression (2/11/21)	Reference values
White blood cell (10^3^/µL)	4.8	14.65	4.6–10.2
Red blood cell (10^6^/µL)	3.66	1.63	4.04–5.48
Hemoglobin (g/dL)	11.1	5.3	12.2–16.2
Hematocrit (%)	34.4	19.5	37.7–47.9
Mean corpuscular volume (fL)	94.0	119.6	80–97
Mean corpuscular hemoglobin (pg)	30.3	32.5	27–31.2
Sedimentation rate (mm/hour)	-	35	0–20
Mean corpuscular hemoglobin concentration (g/dL)	32.3	27.2	31.8–35.4
Platelet count (10^3^/µL)	118	58	142.0–424.0
Mean platelet volume (fL)	9.9	10.1	9.4–12.4
Red cell distribution width (%)	20.0	28.3	11.6–14.6
Nucleated red blood cells (/100 red blood cells)	0.8	13.4	0–0.2

## Discussion

The lack of reported cases of ANKL has contributed to the challenges in making a prompt diagnosis of the disease. Furthermore, its diagnosis is complicated by the absence of standard immunophenotypic and molecular characteristics, as well as the common presence of only a small number of neoplastic NK cells in the bone marrow [5].

In our patient, initial bone marrow aspiration/biopsy was concerning for an EBV infection, but there were no initial indications for a diagnosis of leukemia, lymphoma, or the possibility of a metastatic tumor. Although an abnormal proportion of 5% of the CD2+/CD8+/CD56+ NK cells was seen in the marrow, the significance of such cells was initially uncertain. The bone marrow was subsequently reviewed for other infectious causes, including *Bartonella *and toxoplasmosis, but the results were negative. Afterward, cervical lymph node biopsy results revealed a given diagnosis of EBV+ lymphoproliferative disorder (LPD) versus lymphoma. The lymph node biopsy was sent to the NIH for a second review which concluded a diagnosis of ANKL. The diagnosis was primarily based on immunostaining analysis. The analysis was positive for cytoplasmic CD3, CD2, and CD56, pathognomonic markers for ANKL [9]. The differential diagnoses included EBV+ T-cell lymphoma of childhood. However, the immunophenotype presented above favored an NK-cell over a T-cell derivation of the disease. Initially, the plan was to administer a modified SMILE or P-GEMOX regimen instead of a CHOP-based regimen. However, the disease had progressed to the point where chemotherapy regimens were sidelined due to the possibility of decompensation before the transfer of care to a facility offering stem cell transplants could be conducted. The patient continued to remain critically unstable and succumbed to multiorgan failure before any treatments could be initiated.

The EBV positivity of our patient allowed us to quickly include an EBV+ lymphoproliferative disorder of the NK or T-cell type of childhood into our differentials. However, the lack of standardized diagnostic molecular/immunophenotypic characteristics of the disease was the greatest challenge in making a diagnosis. Had the patient been diagnosed sooner, more aggressive interventions may have been used to hinder disease progression. There have been reports where L-asparaginase monotherapy has been successful in ANKL patients with severe liver dysfunction at the time of diagnosis [10]. After this therapy and the subsequent improvement in liver function, SMILE therapy could then be implemented as well. L-asparaginase-containing regimens and subsequent allogeneic stem cell transplantation has been shown to extend survival in young adult patients, even when the stem cell transplantation was performed in patients who did not have a complete response [11]. Allogeneic stem cell transplantation could have been used because better outcomes are associated with this type of treatment [10].

The known diagnostic difficulties associated with this rare neoplasm underscore the crucial need for further case documentation and investigation on NK-cell lymphoblastic leukemia/lymphomas. Moreover, there is a timely need to narrow down the diagnostic criteria of the malignancy to improve and create a more universal approach to managing this disease.

## Conclusions

This study reports the diagnosis of ANKL in a pediatric patient. The patient had a prior EBV infection and a three-week history of progressive symptoms, including fever, weight loss, and hepatosplenomegaly. Within five weeks from the onset of symptoms, the patient experienced multiorgan failure and expired. The rarity of ANKL in pediatric patients, lack of diagnostic characteristics, and the extremely aggressive nature of this disease require further attention so that diagnoses can be made more readily and further therapeutic options can be developed and introduced.
